# Peptide-Functionalized Nanoparticles-Encapsulated Cyclin-Dependent Kinases Inhibitor Seliciclib in Transferrin Receptor Overexpressed Cancer Cells

**DOI:** 10.3390/nano11030772

**Published:** 2021-03-18

**Authors:** Guan Zhen He, Wen Jen Lin

**Affiliations:** 1 School of Pharmacy, College of Medicine, National Taiwan University, Taipei 10050, Taiwan; r07423010@ntu.edu.tw; 2 Drug Research Center, College of Medicine, National Taiwan University, Taipei 10050, Taiwan

**Keywords:** seliciclib, T7 peptide, nanoparticles, TfR-overexpressed cancer cells

## Abstract

Seliciclib, a broad cyclin-dependent kinases (CDKs) inhibitor, exerts its potential role in cancer therapy. For taking advantage of overexpressive transferrin receptor (TfR) on most cancer cells, T7 peptide, a TfR targeting ligand, was selected as a targeting ligand to facilitate nanoparticles (NPs) internalization in cancer cells. In this study, poly(d,l-lactide-co-glycolide) (PLGA) was conjugated with maleimide poly(ethylene glycol) amine (Mal-PEG-NH_2_) to form PLGA-PEG-maleimide copolymer. The synthesized copolymer was used to prepare NPs for encapsulation of seliciclib which was further decorated by T7 peptide. The result shows that the better cellular uptake was achieved by T7 peptide-modified NPs particularly in TfR-high expressed cancer cells in order of MDA-MB-231 breast cancer cells > SKOV-3 ovarian cancer cells > U87-MG glioma cells. Both SKOV-3 and U87-MG cells are more sensitive to encapsulated seliciclib in T7-decorated NPs than to free seliciclib, and that IC_50_ values were lowered for encapsulated seliciclib.

## 1. Introduction

Cyclin-dependent kinases (CDKs) are a group of threonine/kinases and play regulatory roles in cell cycle or transcription, which requires binding of subunits known as cyclins. Nevertheless, among 21 CDKs, only a certain subset of the CDK/cyclin complex is directly involved in driving the cell cycle, namely, interphase CDKs (CDK2, CDK4 and CDK6) and a mitotic CKD (as known as CDK1) [1]. The development of cancer is characterized by mysregulated CDKs, which contribute to cell cycle defects including unscheduled proliferation, genomic instability and chromosomal instability. In addition, many cancers are uniquely dependent on specific CDKs and, hence, selectively sensitive to inhibition of them. CDK2 is a core cell-cycle component that is essentially active from the late G1-phase and throughout the S-phase; otherwise, CDK2 is expressed at a lower level in most normal tissues [2]. The rationale to target CDK2 for treatment of malignancies includes the indispensable role of CDK2 in proliferation and overexpression of its binding partners, cyclin A or E in several cancers, such as ovary cancer, breast cancer and glioma etc. [3,4,5,6]. Moreover, overexpression of cyclin E has been reported to correlate with the tumor formation in mice and poor prognosis in patients with different cancer types [7]. Owing to the exclusivity of cyclin E for CDK2 and its deregulation in some cancers, CDK2 is an attractive target in cancer therapy.

Seliciclib, also known as (R)-roscovitine or CYC202, is a broad range purine analog inhibitor, which mainly inhibits CDK1, CDK2, CDK5, CDK7 and CDK9 instead of CDK4 and CDK6. Preclinical studies have shown great anti-cancer potential of seliciclib [8,9,10,11,12]. However, clinical trials showed limited benefits in a series of cancer therapies [13,14]. This might be attributed to dependence of CDKs in different stages of tumor development and its rapid metabolism, which limited the maintenance of drug concentration within therapeutic window [15].

In addition to active pharmaceutical ingredients, nanomedicine provides supportive components which improve drug bioavailability as well as aid in drug protection, site-specific activation and cellular uptake [16]. The modification of nanoparticles’ (NPs) surfaces to actively target the overexpressed biomolecules on the surface of a tumor provides specific binding and more efficient internalization of a drug through receptor-mediated endocytosis [17]. Cell-penetrating peptides (CPPs) and short-chain peptides as targeting ligands have been applied to facilitate nanocarriers crossing the blood brain barrier (BBB) and target to glioblastoma as well [18]. CPPs are defined as short chain peptides (no more than 30 amino acids) possessing ability not only to translocate themselves into cells but facilitate the cargo complex entering the targeting sites. The applications of CPPs involve fields of inflammation, central nervous system disorders, ocular disorders and cancer treatment [19]. In the strategy against tumor development, CPPs play an important role in circumvention of the barrier constructed by the tumor and its microenvironment. The application of CPPs in cancer therapy has drawn lots of attention in areas including triple-negative breast cancer, ovarian cancer, colorectal cancer etc. [20,21,22]. Recently, the cationic Tat-peptide modified nanoformulations have been demonstrated to deliver antiviral drugs as well as vaccines for treatment of SARS-CoV-2 infections [23].

Transferrin receptor (TfR) is a 180 kDa membrane glycoprotein, which can import iron by binding transferrin. TfR is classified into two subtypes, TfR1 (known as CD71) and TfR2. TfR1 is a homodimeric type II transmembrane glycoprotein expressed ubiquitously on the surface of most cells while TfR2 is mainly expressed in the liver. TfR1 is expressed on malignant cells at levels about 100-fold higher than those on normal cells, and its expression can be correlated with either tumor stage or cancer progression [24]. Targeting TfR of cancer cells promotes the delivery of therapeutic agents and blocks the natural function of the receptor leading to cancer cell death [25]. T7 peptide (HAIYPRH), composed of seven peptides, has been reported to specifically bind to TfR with high affinity (Kd of 10 nM). Due to different binding site from Tf, endogenous Tf will not compete with the uptake of T7 peptide-modified nanocarriers. Meanwhile, Tf facilitating T7 uptake in vivo has been confirmed which attracts T7 peptide being applied in a cancer-targeting drug delivery system [26,27,28] In this study, we took advantage of NPs and targeting ability of T7 peptide to facilitate seliciclib uptake by cancer cells and achieve a better cytotoxicity effect in TfR-overexpressed cancer cells.

## 2. Materials and Methods

### 2.1. Materials

1-(3-Dimethylaminopropyl)-3-ethylcarbodiimide hydrochloride (EDC), *N*-ethyldiisopropylamine (*N,N*-diisopropylethlamine) (DIEA, 99%), and thiazolyl blue tetrazolium bromide (MTT, 98%) were from Alfa Aesar (Echo Chemical Co., Ltd., Heysham, UK). *N*-hydroxysuccinimide (NHS, 98%) and poly(vinyl alcohol) (PVA, 88% hydrolyzed, 20,000–30,000 g/mol) were from Acros Organics Co., Inc. (Fair Lawn, NJ, USA). Poly(d,l-lactide-co-glycolide) 50:50 (PLGA, ~52,000 g/mol) was from Evonik Industries (Birmingham, AL, USA). Maleimide poly(ethylene glycol) amine (Mal-PEG-amine, 5000 g/mol) was provided by Hunan Hua Teng Pharmaceutical Co., Ltd. (Merelbeke, Belgium). FITC-NHS (MW 473.4 g/mol) was from Thermo Fisher Scientific Inc. (Hudson, NH, USA). FITC-Cys-T7 peptide (1498.71 g/mol, FITC-Cys-His-Ala-Ile-Tyr-Pro-Arg-His-OH) was from Kelowna International Scientific Inc. (Taipei, Taiwan). Anti-human CD71 (transferrin receptor) monoclonal antibody, allophycocyanin (APC) and mouse IgG1 kappa Isotype Control APC were from eBioscience, Inc. (Vienna, Austria). Seliciclib (purity > 99%) was from LC Laboratories (Woburn, MA, USA). A549, MDA-MB-231, SKOV-3 and U87-MG cell lines were from Bioresource Collection and Research Center (Hsinchu, Taiwan).

### 2.2. Synthesis and Characterization of Poly(d,l-Lactide-Co-Glycolide)-Poly(Ethylene Glycol) (PLGA-PEG)-Maleimide Copolymer

PLGA-PEG-maleimide was synthesized by two steps. In the first step, activation of PLGA to PLGA-NHS was performed based on previous method with modification which was subsequently conjugated with NH_2_-PEG-maleimdie [29,30,31]. Briefly, PLGA-NHS was reacted with NH_2_-PEG-maleimide (molar ratio 1:2) in chloroform at room temperature for 24 h in dark. The synthesized PLGA-PEG-maleimide was precipitated with cold methanol/ether co-solvent (1:4 *v*/*v*) and centrifuged under 4000 rpm at 4 °C for 10 min. The precipitate was re-dissolved in chloroform and precipitated by co-solvent three times. Finally, the product was dried in vacuum desiccator for 24 h. The yield and the molar mass were determined. The molar mass of PLGA-PEG-maleimide was determined by size exclusion chromatography (SEC) equipped with a refractive index detector (RI 2031 Plus, Jasco, Tokyo, Japan) and a Styragel^®^ HR 4E column (7.8 mm × 300 mm, Waters, Milford, MA, USA). The mobile phase was high-performance liquid chromatography (HPLC)-graded chloroform and the flow rate was set at 1 mL/min at 35 °C. The copolymer was dissolved in chloroform and filtered through a 0.22 µm polytetrafluoroethylene (PTFE) syringe filter prior to injection. The polystyrene standards were used to construct the calibration curve by plotting the logarithm of the nominal molar mass versus the retention time. The molar mass of PLGA-PEG-maleimide was then calculated based on the calibration curve. The PEGylation efficiency was determined by Equation (1) based on the indicating peaks of PLGA-PEG-maleimide shown in ^1^H nuclear magnetic resonance (NMR) spectrum.

(1) $$\mathrm{PEGylation}\;\mathrm{efficiency}\;(\mathrm{mol}\%)\;=\;\frac{\frac{Area\;\left(3.62\;ppm\right)}{4\;\times \;\frac{MW\;of\;PEG}{MW\;of\;EG\;monomer}}}{\frac{Area\;\left(1.55\;ppm\right)\;+\;Area\;\left(4.80\;ppm\right)\;+\;Area\;\left(5.20\;ppm\right)}{6\;\times \;\frac{MW\;of\;PLGA}{MW\;of\;\left(LA\;monomer\;+\;GA\;monomer\right)}}}\times 100\%$$

### 2.3. Preparation and Characterization of Seliciclib-Loaded Nanoparticles (NPs)

PLGA-PEG-maleimide copolymer was used to prepare NPs for encapsulation of seliciclib. The single emulsion solvent evaporation method was applied to prepare seliciclib-loaded NPs (seliciclib@NPs) [32]. Briefly, seliciclib and copolymer (1:5 *w*/*w*) were dissolved in dichloromethane, and seliciclib-copolymer mixture was added into pH 7.4 phosphate-buffered saline (PBS) containing 0.5% polyvinyl alcohol solution (O/W 1:10 *v*/*v*) under sonication in an ice bath followed by magnetic stir for 4 h. The residual organic solvent was eliminated by rotary evaporator under reduced pressure at 30 °C. The seliciclib@PLGA-PEG-maleimide NPs (seliciclib@PPM NPs) were collected after centrifugation and washed with water twice. To prepare seliciclib loaded T7 peptide-conjugated NPs (seliciclib@PPM NPs-Cys-T7), seliciclib@PPM NPs and Cys-T7 peptide (molar ratio 1:2) were incubated in pH 7.4 PBS for 2 h and then collected after centrifugation followed by lyopilization [33]. The yields of seliciclib-loaded NPs were calculated. The peptide conjugation efficiency was calculated by using Equation (2) where the fluorescence of fluorescein isothiocyanate (FITC)-labeled T7 peptide was determined.

(2) Cys-T7 peptide conjugation ratio (mol%) = Conc. of FITC labeled Cys-T7 peptideMW of FITC labeled Cys-T7 peptide (1498.71 g/mol)Conc. of FITC labeled PLGA-PEG-maleimide-Cys-T7MW of FITC labeled PLGA-PEG-maleimide-Cys-T7 (59,700 g/mol)

The particle size and zeta potential were determined by zetasizer (Nano-ZS90, Malvern Instruments, Worcestershire, UK). The amount of seliciclib encapsulated by NPs was determined by HPLC detected at 290 nm. The drug loading (DL) and encapsulation efficiency (EE) were calculated. The morphology of NPs was observed by transmission electron microscope (TEM) (Hitachi H-7650, Hitachi High-Technologies Corporation, Tokyo, Japan). The NPs suspension was dripped on copper grids (300 mesh Formvar/carbon coated) and pended for 30 s. The excess solution was removed by filter paper. The phosphotungstic acid solution (2% *w*/*v*) was added and placed for another 30 s to stain the NPs. After removing the excess fluid, the sample was proceeded for observation with 50 K and 400 K magnifications. For the stability study, the freshly prepared seliciclib@NPs was mixed with sucrose solution followed by lyophilization overnight. The lyophilized seliciclib@NPs was stored at −20 °C, and the samples were collected at day 0, 7, 14, 21 and 28 after lyophilization. Each sample was resuspended in deionized water, and the particle size, polydispersity index (PdI), and zeta potential were measured.

In vitro release of seliciclib from seliciclib@PPM NPs and seliciclib@PPM NPs-Cys-T7 was conducted in pH 7.4 PBS release medium at 37 °C. The lyophilized seliciclb@NPs were loaded in the dialysis bag (cut off MW 12,000–14,000 g/mol) and immersed in release medium under shaking at 100 rpm. The medium was withdrawn at each specific time point, and same volume of fresh release medium was added. The collected samples were subjected to centrifugation and the concentration of seliciclib in the medium was quantified by the HPLC method.

### 2.4. Determination of Transferrin Receptor Expression Level

In order to confirm the expression level of transferrin receptor (TfR) on the surface of cancer cells, the APC-conjugated anti-human CD71 monoclonal antibody and mouse IgG1 kappa isotype control were applied. Briefly, the cells were trypsinized from a 10 cm dish and collected, and the cell pellet was resuspended in staining buffer. The anti-human CD71 antibody or mouse IgG1 kappa isotype was added into the tube and reacted for 1 h followed by centrifugation and washing with staining buffer for three times. The cell pellet was then resuspended in staining buffer and analyzed by FACSCalibur (Becton Dickinson, Franklin Lakes, NJ, USA). A total of 10,000 events were analyzed, and the upper limit of the IgG isotype control was set no more than 1% of the events with non-specific binding. The M1 gated (%) and relative mean fluorescence intensity (relative MFI) calculated by Equation (3) were obtained.

(3) $$\mathrm{Relative}\;\mathrm{MFI}=\frac{\left({\mathrm{MFI}}_{\mathrm{Anti}-\mathrm{CD}71}\;-\;{\mathrm{MFI}}_{\mathrm{IgG}1\;\mathrm{isotype}}\right)}{{\mathrm{MFI}}_{\mathrm{no}\;\mathrm{treatment}\;\left(\mathrm{unstained}\right)}}$$

### 2.5. Cellular Uptake Study

The cellular uptake of peptide-conjugated PPM NPs-Cys-T7 was investigated, and the peptide-free PLGA-PEG NPs (PP NPs) were served as the control group. The amount of NPs uptake by cells was determined by mean fluorescence intensity (MFI) derived from fluorescence probe FITC. MDA-MB-231 cells, SKOV-3 cells, U87-MG cells or A549 cells were uniformly seeded in 24-well plates at a density of 2 × 10^5^ cells/well in medium (McCoy’s 5A medium for SKOV-3 cells; Dulbecco’s Modified Eagle medium (DMEM) for others) containing 10% bovine growing serum and 1% PSA, and incubated for 24 h. The PP NPs and PPM NPs-Cys-T7 were added and incubated in 5% CO_2_ at 37 °C for 2 h. The cells were washed with PBS for three times and trypsinized followed by centrifugation. Finally, the cells were collected and the fluorescence intensity was measured by flow cytometer (BD FACSCalibur Becton Dickinson, Franklin Lakes, NJ, USA). A total of 10,000 events were analyzed for each sample, and the cellular uptake efficiency in terms of relative mean fluorescence intensity was calculated as follows:

(4) $$\mathrm{Cellular}\;\mathrm{uptake}\;\mathrm{efficiency}\;=\;\frac{{\mathrm{MFI}}_{\mathrm{PPM}\;\mathrm{NPs}-\mathrm{Cys}-T7}-{\mathrm{MFI}}_{\mathrm{PP}\;\mathrm{NPs}}}{{\mathrm{MFI}}_{\mathrm{non}-\mathrm{treatment}}}$$

### 2.6. Fluorescence Microscopy

MDA-MB-231, SKOV-3, U87-MG and A549 cells were seeded at a density of 2 × 10^5^ cells/well on coverslip in 6-well plates. After 24-h incubation, the medium was removed, and PBS was added to wash the cells, further replaced by a final concentration of 1 mg/mL of NPs in free DMEM medium except McCoy’s 5A medium for SKOV-3 cells. After incubation at 37 °C in 5% CO_2_ for 2 h, cells were washed with cold PBS and fixed with cold methanol. After washing with PBS, the nucleus was subsequently stained with 4′,6-diamidino-2-phenylindole (DAPI), followed PBS washing cycle. Finally, coverslip was covered on the slide with Fluoromount gel. The sample slides were imaged by a fluorescence microscope (Zeiss AxioImager. A1, Jena, Germany).

### 2.7. Cytotoxicity of Seliciclib@NPs (Nanoparticles)

MDA-MB-231 cells, SKOV-3 cells, U87-MG cells or A549 cells were uniformly seeded in 96-well plates at a density of 1 × 10^4^ cells/well in medium (McCoy’s 5A medium for SKOV-3 cells; DMEM medium for others) containing 10% bovine growing serum and 1% PSA. After 24 h incubation, the medium was replaced with various concentrations of free seliciclib or seliciclib@NPs (1–50 µg/mL). The cells were further incubated in 5% CO_2_ at 37 °C for 48 h. Subsequently, the MTT solution was added into each well for another 4-h incubation. Finally, the supernatant was removed, and dimethyl sulfoxide (DMSO) was added to dissolve the formazan crystal. The absorbance was measured at 570 nm and 690 nm by a microplate reader (SpectraMax Paradigm, Molecular Devices, San Jose, CA, USA) and cytotoxicity was interpreted as follows:

(5) $$\mathrm{Cytotoxicity}\;\left(\%\right)\;=\;\left[1-\frac{{\left[OD_{570\;nm}-OD_{690\;nm}\right]}_{sample}}{{\left[OD_{570\;nm}-OD_{690\;nm}\right]}_{control}}\right]\;\times \;100\%$$

### 2.8. Statistical Analysis

All statistical analysis was conducted by SigmaPlot 12.5 (Softhome International, Inc., Taipei, Taiwan). One-way analysis of variance (ANOVA) and unpaired Student’s *t*-test were applied, and the statistical significance was defined as *p* < 0.05.

## 3. Results and Discussion

### 3.1. Characterization of PLGA-PEG-Maleimide Copolymer

**Figure 1:**
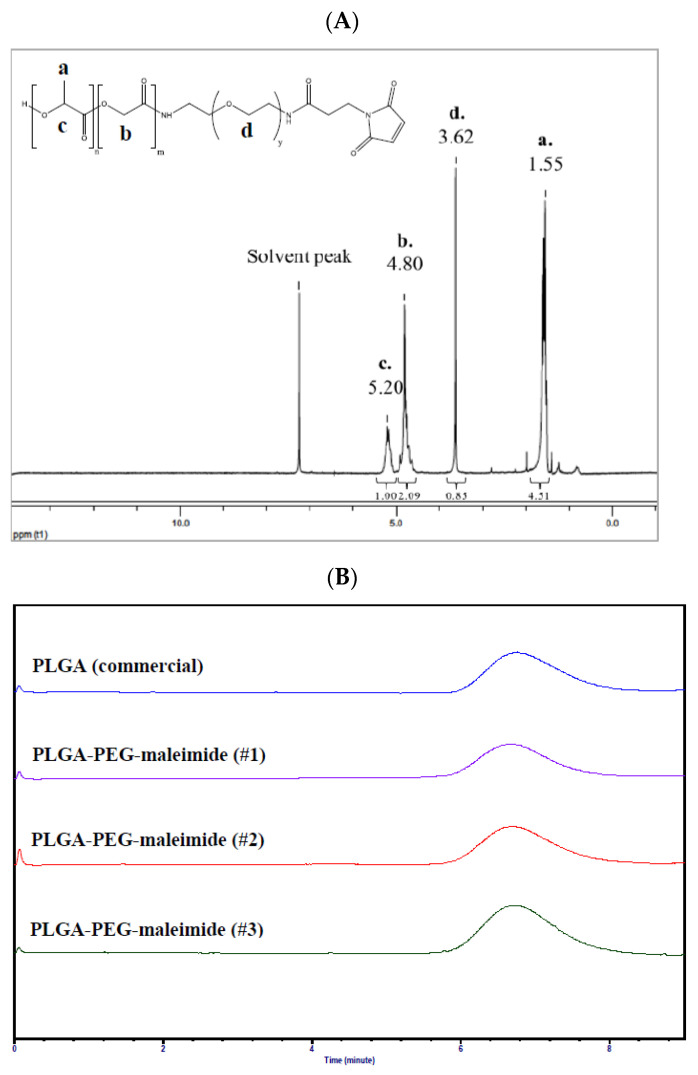
(**A**) ^1^H nuclear magnetic resonance (NMR) spectrum and (**B**) size exclusion chromatograms of poly(d,l-lactide-co-glycolide)-poly(ethylene glycol)-maleimide (PLGA-PEG-mal, n = 3).

Figure 1A illustrates the ^1^H NMR spectrum of PLGA-PEG-maleimide. The signals at δ 1.55 ppm, δ 4.80 ppm, and δ 5.20 ppm derived from PLGA represent -CH3 protons of lactide (a), -CH2 protons of glycolide (b), and -CH proton of lactide (c), respectively. On the other hand, the signal at δ 3.62 ppm represents -CH2- protons of PEG-maleimide (d). All of these indicating peaks in the ^1^H NMR spectrum imply the successful conjugation of PEG-maleimide onto PLGA. The yield of synthesized PLGA-PEG-maleimide was 53.4 ± 3.4%. The molar mass of PLGA-PEG-maleimide was determined by size exclusion chromatography (SEC) (Figure 1B). The weight-average molar mass (M_w_), number-average molar mass (M_n_), and dispersity of synthesized PLGA-PEG-maleimide were 59,700 ± 2600 g/mol, 32,000 ± 1900 g/mol and 1.87 ± 0.03, respectively. The M_w_ increased approximately 8000 g/mol after conjugation with PEG compared to commercial PLGA, and the PEGylation efficiency was 60.4 ± 4.0 mol%.

### 3.2. Characterization of Seliciclib-Loaded NPs

**Figure 2:**
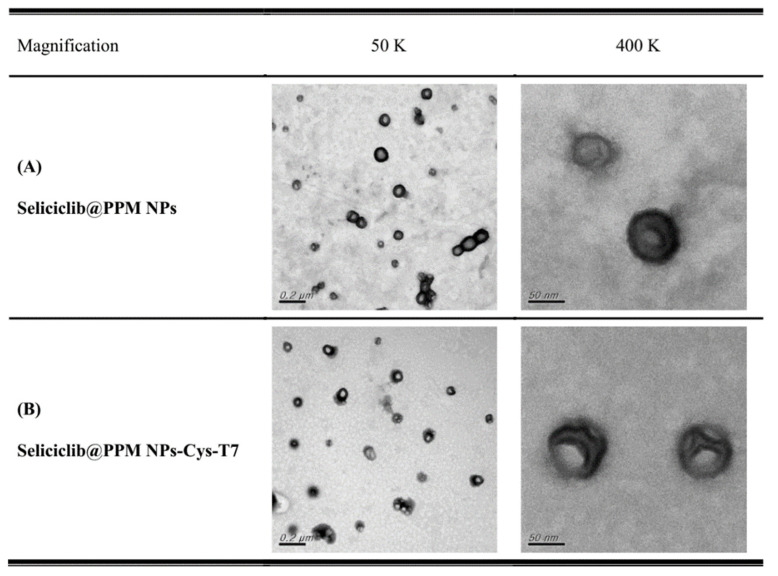
Transmission electron microscope (TEM) images of (**A**) seliciclib@PPM NPs and (**B**) seliciclib@PPM NPs-Cys-T7 with magnification 50 K (scale bar: 200 nm) and 400 K (scale bar: 50 nm).

PLGA-PEG-maleimide NPs (PPM NPs) were prepared followed by functionalized with T7 peptide via maleimide-thiol linkage, and the peptide conjugation efficiency was 26.9 ± 4.8 mol%. The anticancer drug, seliciclib, was encapsulated by peptide-free and peptide-conjugated NPs, respectively, and the characteristics of seliciclib@PPM NPs and seliciclib@PPM NPs-Cys-T7 are summarized in Table 1. The particle sizes of seliciclib@PPM NPs and seliciclib@PPM NPs-Cys-T7 were 115.7 ± 5.5 nm and 127.3 ± 0.7 nm with narrow size distribution (PdI 0.11 ± 0.03 and 0.19 ± 0.03, respectively). The increasing zeta potential from −30.8 ± 9.2 mV to −20.0 ± 4.2 mV was observed after conjugation of T7 peptide. The encapsulation efficiency (EE) of seliciclib was 64.8 ± 3.7% for seliciclib@PPM NPs and 60.0 ± 1.2% for seliciclib@PPM NPs-Cys-T7, and the corresponding drug loading (DL) was 14.9 ± 1.0% and 12.3 ± 0.5%, respectively. The TEM images illustrate these particles were separated with spherical shape (Figure 2). The size of seliciclib@NPs observed in TEM images was approximately 90 nm which was slightly smaller than the ones measured by dynamic light scattering (DLS) method (115.7 nm to 127.3 nm). It might be attributed to the difference in sample preparation process. For DLS method, the NPs suspension was diluted with deionized water followed by measurement directly. In contrast, the NPs sample for TEM imaging was subjected to the drying process to retain the particles on the mesh for observation. This dehydration process induced the shrinkage of outer PEG hydrophilic layer and smaller size of particles was observed.

### 3.3. Stability and Release of Seliciclib@NPs

**Figure 3:**
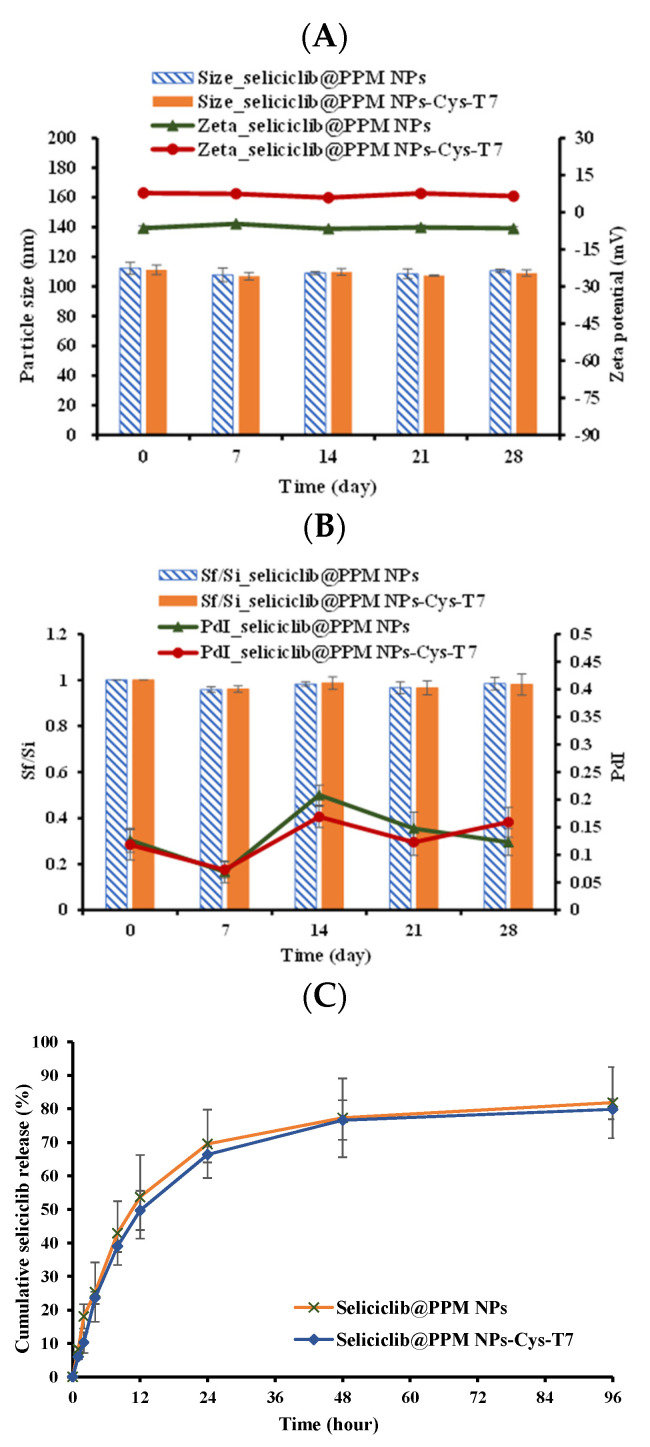
Stability of lyophilized seliciclib@NPs for 28 days. (**A**) Particle sizes (bar) and zeta potentials (line). (**B**) Final to initial size ratio (S_f_/S_i_) (bar) and PdIs (line). (**C**) Cumulative release of seliciclib from seliciclib@NPs in pH 7.4 phosphate-buffered saline (PBS) release medium at 37 °C. (n = 3, mean ± SD).

The lyophilized NPs were stored at −20 °C for 28 days. The samples were collected at specific time points and re-dispersed in deionized H_2_O for particle size, PdI, and zeta potential measurement. All seliciclib@PPM NPs and seliciclib@PPM NPs-Cys-T7 maintained their particle size during storage (Figure 3A) and the final to initial size ratios (S_f_/S_i_) were within 5% (ranging from 0.96 to 0.99) with PdIs below 0.25 (Figure 3B) indicating no aggregation occurred. In addition, there was no obvious change in zeta potentials of NPs after lyophilization and reconstitution as well (Figure 3A). All of these results implied the great stability of lyophilized seliciclib@PPM NPs and seliciclib@PPM-Cys-T7 NPs in these storage conditions following reconstitution with deionized water.

The release of seliciclib from NPs under simulated physiological conditions was demonstrated in pH 7.4 PBS. Figure 3C illustrates the in vitro release of seliciclib from seliciclib@PPM NPs and seliciclib@PPM NPs-Cys-T7 in pH 7.4 PBS release medium at 37 °C. There were 81.9 ± 10.7% and 79.9 ± 3.0% of drug released from seliciclib@PPM NPs and seliciclib@PPM NPs-Cys-T7, respectively, within 96 h, and the corresponding t_50_ values (the time for 50% of seliciclib released) were 12.8 ± 4.6 and 13.2 ± 0.8 h. These results indicate that the conjugation of peptide onto NPs did not affect seliciclib release and the similar t_50_ values were observed in seliciclib@PPM NPs and seliciclib@PPM NPs-Cys-T7.

### 3.4. Cellular Uptake of NPs

**Figure 4:**
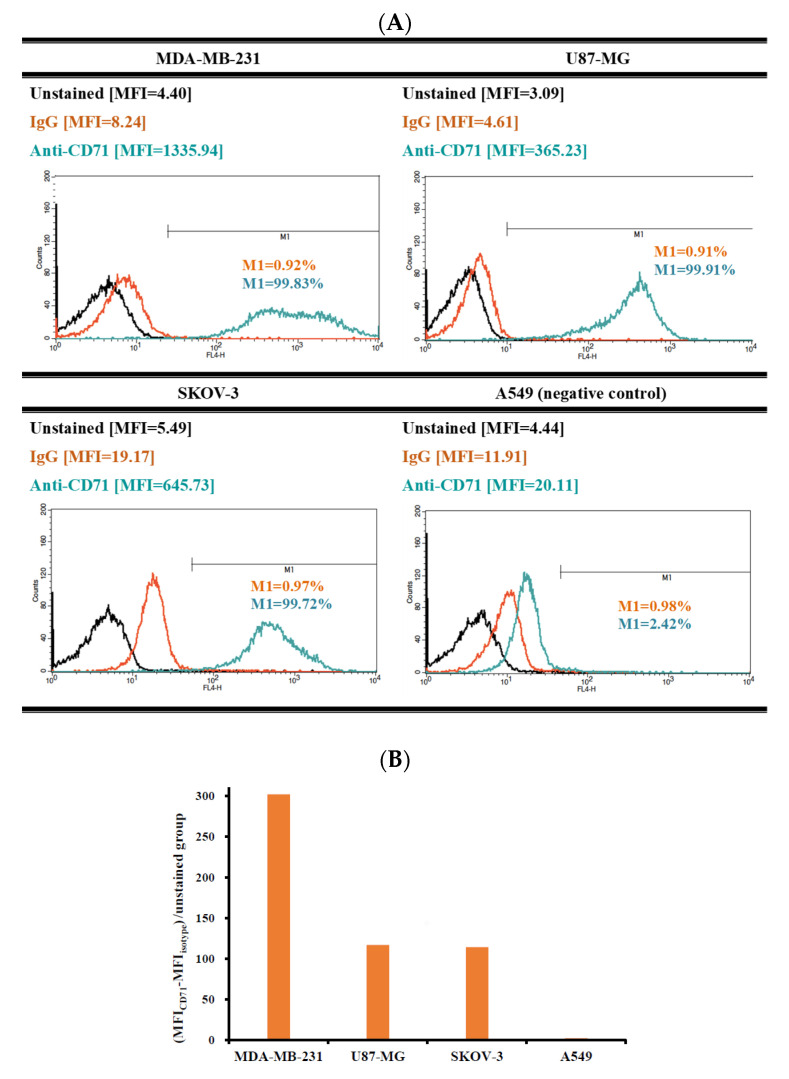
(**A**) The cytometric histograms of various cancer cells stained with anti-human CD71 antibody (green line) and isotype IgG control (orange line), the black line indicated the group without any treatment. (**B**) Transferrin receptor (TfR) expression levels expressed in relative mean fluorescence intensity (MFI) calculated by Equation (3).

Since TfR plays an important role in dominating cellular uptake of T7 peptide-modified NPs, the TfR expression levels in MDA-MB-231 breast cancer cells, SKOV-3 ovarian cancer cells, U87-MG glioma cells, and A549 non-small cell lung cancer cells were determined first. The degree of transferrin receptor expression was defined as low expression with 0–30% of M1 gated, medium expression with 30–60% of M1 gated and high expression with 61–100% of M1 gated [34]. Figure 4A shows MDA-MB-231, U87-MG, and SKOV-3 cancer cells had a high expression of TfR with 99.83%, 99.91% and 99.72% of M1 gated, respectively. On the other hand, A549 cancer cells had a low expression of TfR with 2.42% of M1 gated. Among three highly TfR-expressive cancer cells, MDA-MB-231 cells exhibited the highest relative MFI of 301.8 compared to 114.1 for SKOV-3 cells and 116.7 for U87-MG cells (Figure 4B).

**Figure 5:**
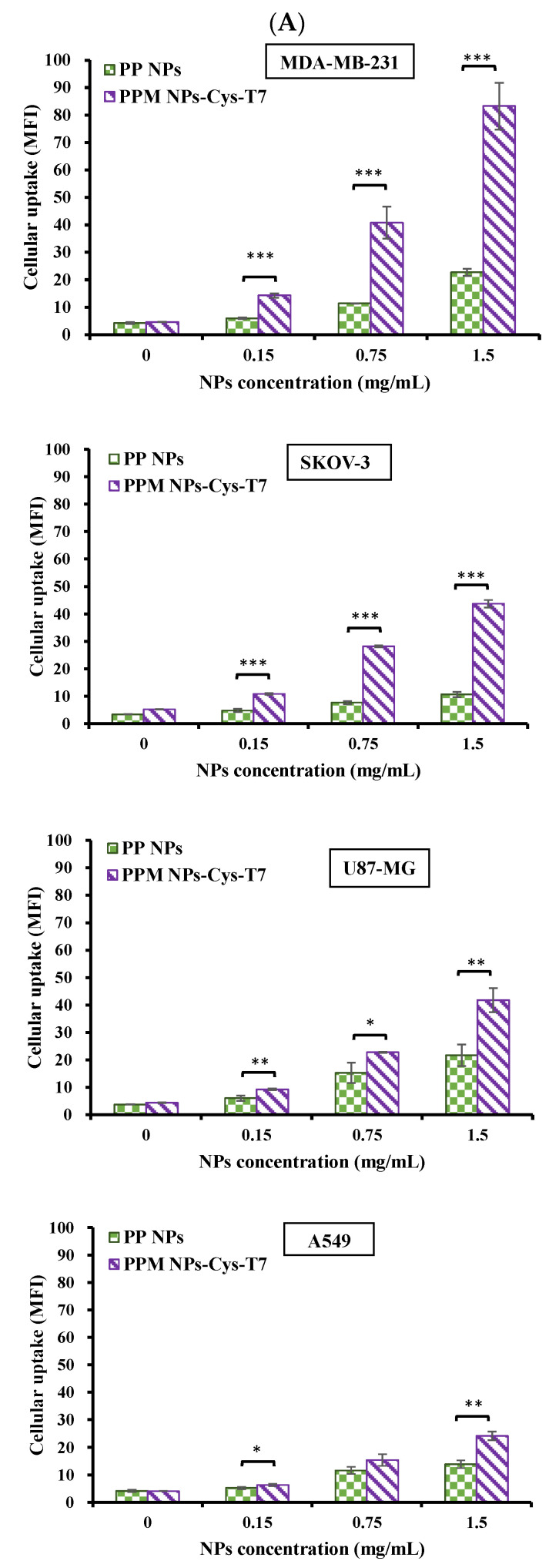

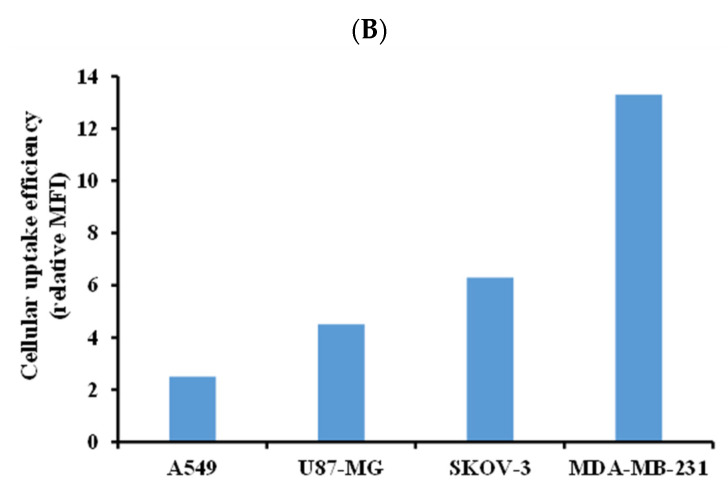
(**A**) Cellular uptake of PLGA-PEG (PP NPs) and PLGA-PEG-maleimide (PPM) NPs-Cys-T7 in four cancer cell lines under 5% CO_2_ at 37 °C for 2 h analyzed by flow cytometer (n = 3, mean ± SD, * *p* < 0.05, ** *p* < 0.01, *** *p* < 0.001, compared to PP NPs). (**B**) Cellular uptake efficiency of PPM NPs-Cys-T7 (relative MFI calculated by Equation (3)) at 1.5 mg/mL in four cancer cell lines.

The uptake of PP NPs and PPM NPs-Cys-T7 in various cancer cell lines for 2 h was analyzed by flow cytometer and the mean fluorescence intensity (MFI) was determined. Figure 5A shows that, generally, the uptake of T7-modified NPs (PPM NPs-Cys-T7) was elevated as compared to peptide-free NPs (PP NPs) in all cancer cells, especially in highly TfR-expressive MDA-MB-231 cells. In contrast, A549 cells were used as a negative control of TfR-expression and displayed the lowest MFI among these cancer cell lines. These results indicated the improvement of NPs uptake in TfR-overexpressed cancer cells was through T7 peptide. The cellular uptake efficiency, in terms of relative MFI calculated by Equation (4), of PPM NPs-Cys-T7 at 1.5 mg/mL in four cancer cell lines was further displayed in Figure 5B. Herein MDA-MB-231 cells exerted the highest cellular uptake efficiency followed by SKOV-3, U87-MG and A549 cells.

**Figure 6:**
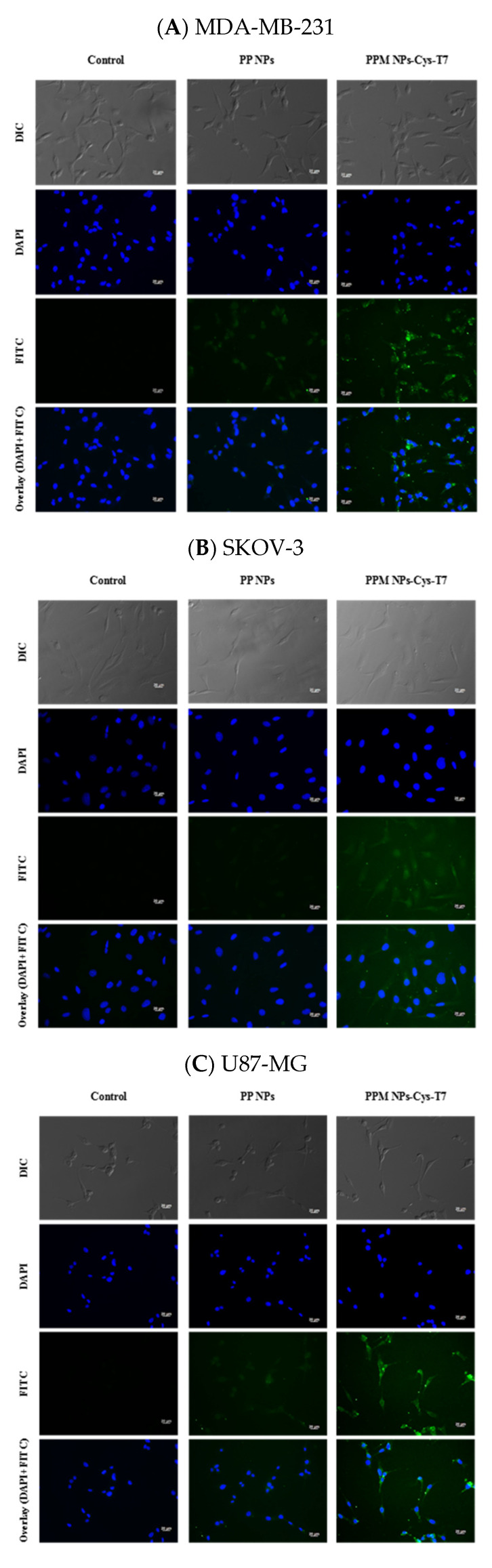

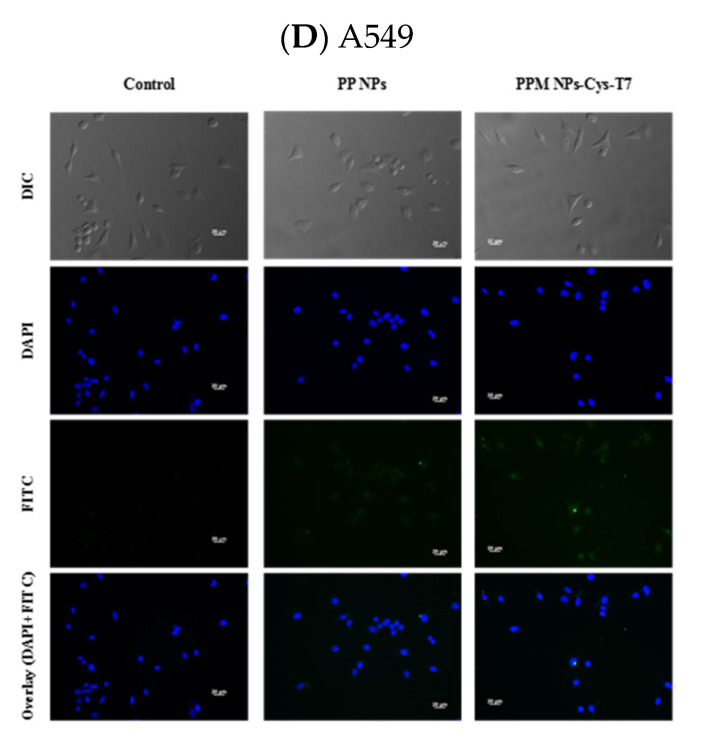
Fluorescence microscopic images of (**A**) MDA-MB-231, (**B**) SKOV-3, (**C**) U87-MG and (**D**) A549 cells treated with free medium only (left) and FITC-labeled PP NPs (middle) and PPM NPs-Cys-T7 (right). The blue spots indicate the nuclei stained with DAPI, and the green signals present the FITC-labeled NPs. (400×, scale bar 20 µm).

Figure 6 shows the fluorescence microscope images for cellular uptake of FITC-labeled NPs in MDA-MB-231 (Figure 6A), SKOV-3 (Figure 6B), U87-MG (Figure 6C), and A549 cells (Figure 6D), respectively. The cell nuclei, presented as blue, were stained with DAPI, and the green signals were derived from the FITC labeled NPs. The fluorescence intensity of PP NPs was very weak no matter in TfR-high expressed cancer cells (e.g., MDA-MB-231, SKOV-3, and U87-MG) or TfR-low expressed A549 cells. The images show stronger fluorescence intensity for peptide-conjugated PPM NPs-Cys-T7 than for peptide-free PP NPs, especially in highly TfR-expressed cancer cell lines (e.g., MDA-MB-231, SKOV-3, and U87-MG cells), indicating more NPs internalized into TfR-overexpressed cells via functional T7 peptide. However, there was slight difference in fluorescence intensity between PP NPs and PPM NPs-Cys-T7 in negative control A549 cells.

**Figure 7:**
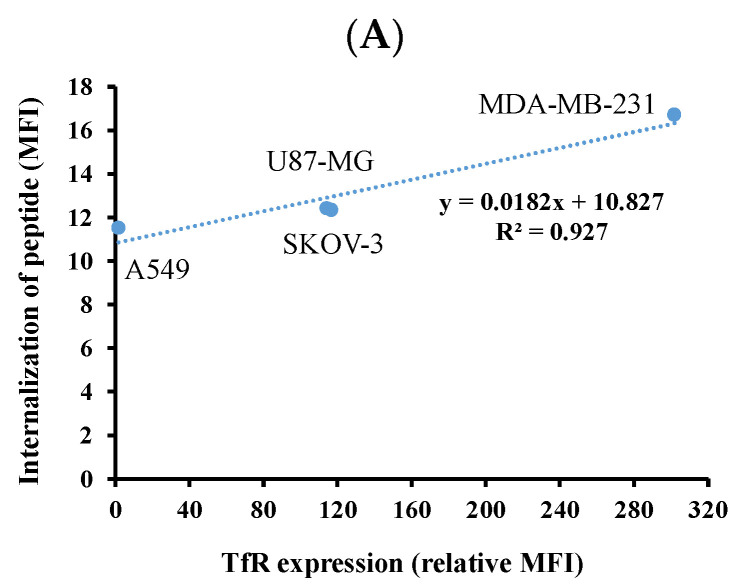

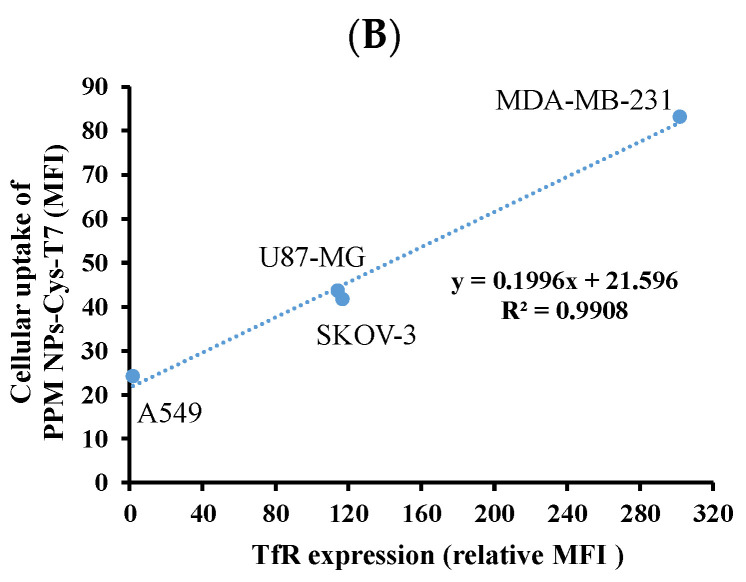
Correlation of TfR expression level and (**A**) internalization of T7 peptide as well as (**B**) cellular uptake of PPM NPs-Cys-T7 in four cancer cell lines.

Since transferrin receptor plays an important role in delivery of T7 peptide-functionalized NPs into tumor cells, the correlation of TfR expression level and internalization of T7 peptide (Figure 7A) as well as cellular uptake of PPM NPs-Cys-T7 (Figure 7B) are further evaluated. It is found that the cellular uptake of PPM NPs-Cys-T7 (y) exerts high correlation to TfR expression level (×) with R^2^ ~ 0.991 in these four cancer cells (Figure 5B). The role of T7 peptide in the presence of NPs or not is worth elucidating. The slope of the correlation line shown in Figure 5 is served as the indicator for utilization of TfR on either internalization of peptide or uptake of peptide-conjugated PPM NPs-Cys-T7. Figure 5A demonstrates the utilization of TfR with the treatment of T7 peptide alone in terms of slope 0.0182, while for the conjugation of T7 peptide onto NPs, the slope of correlation equation increased to 0.1996 (Figure 7B). This implied that PPM NPs-Cys-T7 exerts ten-fold efficient utilization of TfR as compared to T7 peptide alone. This result elucidates the important role of T7 peptide particularly in the presence of NPs. A combination of targeting peptide and nanocarriers allows PPM NPs-Cys-T7 possessing synergistic effect on delivery of seliciclib@NPs via receptor-mediated pathway to induce cancer cell apoptosis. In other words, the correct orientation of T7 peptide on NPs is feasible for recognizing TfR on receptor overexpressed tumor cells resulting in higher cellular uptake efficiency of PPM NPs-Cys-T7.

### 3.5. Cytotoxicity of Seliciclib@NPs

**Figure 8:**
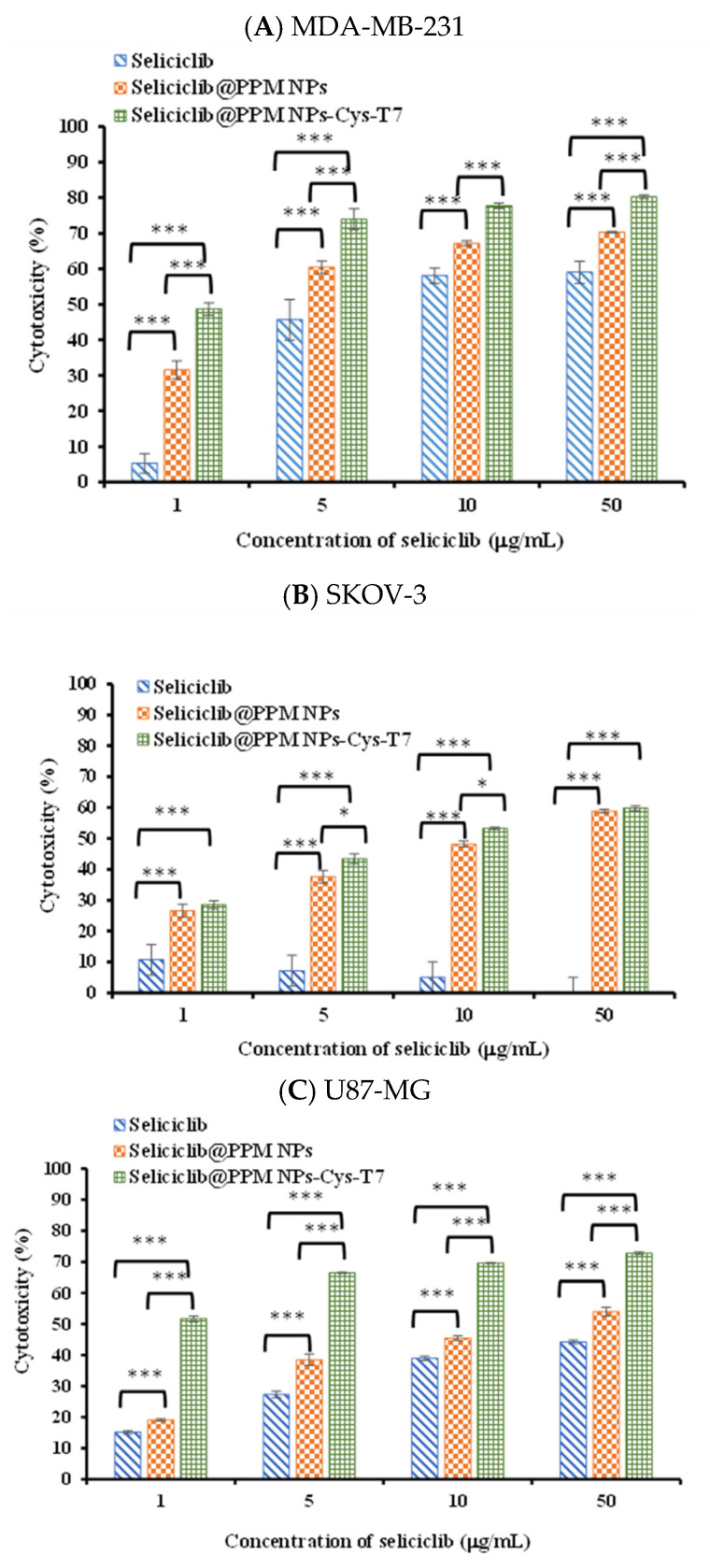

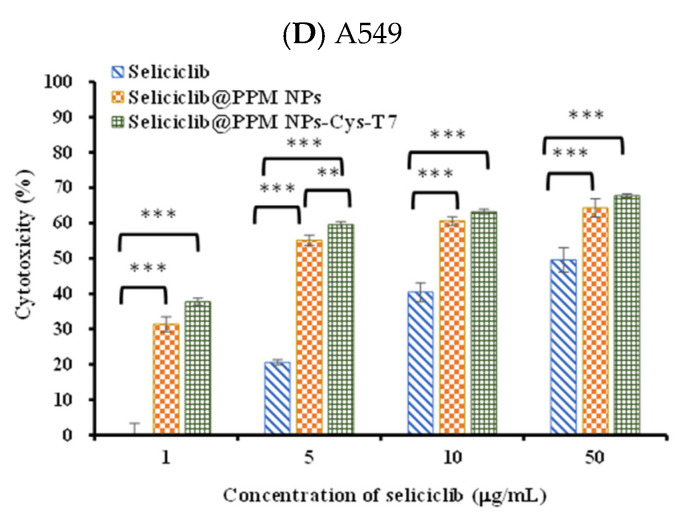
Cytotoxicity of seliciclib, seliciclib@PPM NPs and seliciclib@PPM NPs-Cys-T7 in (**A**) MDA-MB-231, (**B**) SKOV-3, (**C**) U87-MG and (**D**) A549 cells for 48 h. (n = 3, mean ± SD, * *p* < 0.05, ** *p* < 0.01, *** *p* < 0.001).

Figure 8 shows the cytotoxicity of seliciclib, seliciclib@PPM NPs, and seliciclib@PPM NPs-Cys-T7 in MDA-MB-231, SKOV-3, U87-MG, and A549 cells for 48 h, and the corresponding IC_50_ values are summarized in Table 2. MDA-MB-231 cells exhibited high susceptibility to free seliciclib with IC_50_ 3.58 ± 0.91 µg/mL. The IC_50_ values of seliciclib@PPM NPs and seliciclib@PPM NPs-Cys-T7 in MDA-MB-231 cells were 2.49 ± 1.13 µg/mL and 2.03 ± 0.24 µg/mL, respectively, showing a decrease tendency without significant difference from that of free seliciclib. Both SKOV-3 and U87-MG cells were relatively insensitive to seliciclib with IC_50_ > 50 µg/mL. However, the IC_50_ of seliciclib@PPM NPs significantly reduced to 7.09 ± 0.25 µg/mL in SKOV-3 and 4.39 ± 0.27 µg/mL in U87-MG as compared to free seliciclib (^###^ *p* < 0.001), which further lowered to 4.92 ± 0.19 µg/mL and 1.35 ± 0.28 µg/mL respectively by seliciclib@PPM NPs-Cy-T7 (*** *p* < 0.001). This means more efficient delivery of seliciclib by NPs, particularly the peptide-modified NPs into SKOV-3 and U87-MG cancer cells. A549 cells were not sensitive to seliciclib either with IC_50_ > 50 µg/mL. Nevertheless, the IC_50_ of seliciclib@PPM NPs significantly reduced to 3.02 ± 0.50 µg/mL as compared to free seliciclib (^###^ *p* < 0.001) due to enhanced permeability and retention (EPR) effect of NPs. There was no significant difference in IC_50_ between seliciclib@PPM NPs and seliciclib@PPM NPs-Cys-T7 in A549, implying the conjugation of T7 peptide did not affect the cytotoxicity in cancer cells with low TfR expression.

## 4. Conclusions

To the best of our knowledge, this study was the first to encapsulate seliciclib in T7 peptide conjugated PLGA-PEG NPs (seliciclib@PPM NPs-Cys-T7) for cancer therapy. The advantage of NPs and the targeting ability of T7 peptide concurrently enhanced cellular uptake of peptide-conjugated PPM NPs-Cys-T7 in TfR-high expressed cancer cells in order of MDA-MB-231 > SKOV-3 > U87-MG. For seliciclib-loaded NPs, since MDA-MB-231 cells exhibited high susceptibility to free seliciclib, the further effects of NPs and T7 peptide on its cytotoxicity were limited. Instead of low susceptibility to free seliciclib by SKOV-3 and U87-MG cancer cells, the antitumor cytotoxicity in terms of IC_50_ was prominently lowered by seliciclib@NPs particularly the T7 peptide-conjugated seliciclib@PPM NPs-Cys-T7.

## Figures and Tables

**Table 1:** The characteristics of seliciclib@PLGA-PEG-maleimide nanoparticles (seliciclib@PPM NPs) and seliciclib@PPM NPs-Cys-T7.

	Seliciclib@PPM NPs	Seliciclib@PPM NPs-Cys-T7
Particle size (nm)	115.7 ± 5.5	127.3 ± 0.7
PdI	0.11 ± 0.03	0.19 ± 0.03
Zeta potential (mV)	−30.8 ± 9.2	−20.0 ± 4.2
Yield (%)	72.5 ± 3.6	81.3 ± 1.7
EE (%)	64.8 ± 3.7	60.0 ± 1.2
DL (%)	14.9 ± 1.0	12.3 ± 0.5
Peptide conjugation (mol%)	-	26.9 ± 4.8

**Table 2:** The IC_50_ of seliciclib, seliciclib@PPM NPs and seliciclib@PPM NPs-Cys-T7 in four cancer cell lines for 48-h treatment. (n = 3, mean ± SD, ^###^ *p* < 0.001, compared to IC_50_ of seliciclib; *** *p* < 0.001, compared to IC_50_ of seliciclib@PPM NPs).

	Cell Line	MDA-MB-231	SKOV-3	U87-MG	A549
IC_50_ (µg/mL)					
Seliciclib		3.58 ± 0.91	>50	>50	>50
Seliciclib@PPM NPs		2.49 ± 1.13	7.09 ± 0.25 ^###^	4.39 ± 0.27 ^###^	3.02 ± 0.50 ^###^
Seliciclib@PPM NPs-Cys-T7		2.03 ± 0.24	4.92 ± 0.19 ^###,^***	1.35 ± 0.28 ^###,^***	3.09 ± 0.16 ^###^
